# Robot-assisted laparoscopic partial nephrectomy: Current review of the technique and literature

**DOI:** 10.4103/0972-9941.59305

**Published:** 2009

**Authors:** Iqbal Singh

**Affiliations:** Department of Urology, Wake Forest University Medical School and Baptist Medical Centre, Medical Centre Boulevard, Winston Salem - 27157, North Carolina, USA

**Keywords:** Laparoscopic partial nephrectomy, laparoscopic surgery, partial nephrectomy, robot, robot-assisted laparoscopic partial nephrectomy

## Abstract

**AIM:**

To visit the operative technique and to review the current published English literature on the technique, and outcomes following robot-assisted laparoscopic partial nephrectomy (RPN).

**MATERIALS AND METHODS:**

We searched the published English literature and the PubMed^(™)^ for published series of ‘robotic partial nephrectomy’ (RPN) using the keywords; robot, robot-assisted laparoscopic partial nephrectomy, laparoscopic partial nephrectomy, partial nephrectomy and laparoscopic surgery.

**RESULTS:**

The search yielded 15 major selected series of ‘robotic partial nephrectomy’; these were reviewed, tracked and analysed in order to determine the current status and role of RPN in the management of early renal neoplasm(s), as a minimally invasive surgical alternative to open partial nephrectomy. A review of the initial peri-operative outcome of the 350 cases of select series of RPN reported in published English literature revealed a mean operating time, warm ischemia time, estimated blood loss and hospital stay, of 191 minutes, 25 minutes, 162 ml and 2.95 days, respectively. The overall computed mean complication rate of RPN in the present select series was about 7.4%.

**CONCLUSIONS:**

RPN is a safe, feasible and effective minimally invasive surgical alternative to laparoscopic partial nephrectomy for early stage (T_1_) renal neoplasm(s). It has acceptable initial renal functional outcomes without the increased risk of major complications in experienced hands. Prospective randomised, controlled, comparative clinical trials with laparoscopic partial nephrectomy (LPN) are the need of the day. While the initial oncological outcomes of RPN appear to be favourable, long-term data is awaited.

## INTRODUCTION

The widespread use of imaging as a screening modality for various diagnostic and health check ups has led to a rapid increase in the frequency of detection of small, low-grade and early renal tumours; with a vast majority (three-fourth) of them being less than 4 cms in size (T_1a_).[1] The primary goals of management of such renal neoplasm(s) include, maximising local cancer control (by minimising margin positivity and cancer recurrence), preserving renal function (by minimising the warm ischemia time) and minimising patient morbidity/hospital stay (through better haemostasis and lower robotic/console operating times). The current management options include nephron sparing surgery or partial nephrectomy through open or minimally invasive surgery (laparoscopic or robot-assisted laparoscopic ablation, cryoablation, radiofrequency ablation). We review the technique and currently published select series on robot-assisted laparoscopic partial nephrectomy (RPN) that have been reported in English literature to date.

### Robot-assisted transperitonal technique of partial nephrectomy

**Figure 1:**
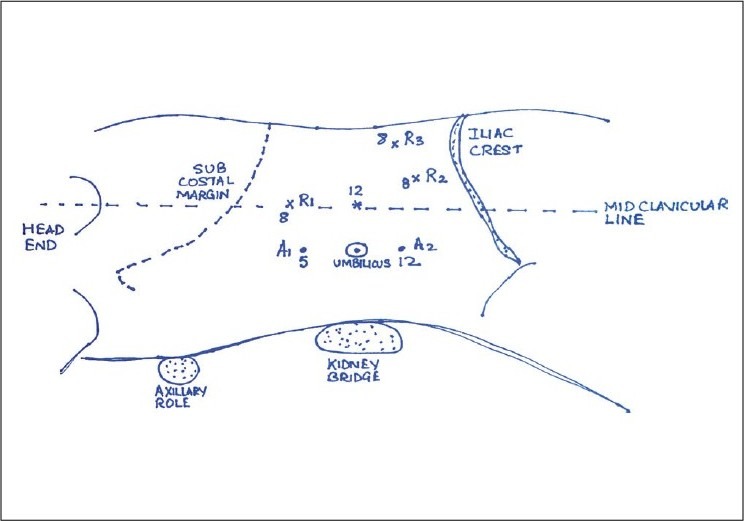
A diagrammatical sketch showing the placement of the ports in a case of left-sided, robot-assisted laparoscopic partial nephrectomy. The 12 mm port site is the site for docking the robotic camera that is placed above the umbilicus, just outside the lateral border of the rectus in the mid-clavicular line. R_1_, R_2_ and R_3_ depict the three 8 mm port sites for the three robotic arms. R_1_ and R_2_ are placed almost on an oblique line, on either side of the primary camera port; the R_3_ port, if used, is placed just above the iliac crest. A_1_ and A_2_ represent the 5 mm and 12 mm assistant ports that are placed, almost equidistant, on either side of the umbilicus in the midline

*(i) Patient positioning*, *port placement and docking*: The procedure is initiated with the patient in the supine position, under general endotracheal anaesthesia. The patient is placed in the modified flank position with an axillary and kidney roll, leg padding/pillows and a padded arm holder for the ipsilateral upper extremity. A high flow, low pressure pneumoperitoneum is obtained with a Veress needle. An incision is made superolateral to the umbilicus (pararectal) for the 12 mm camera port. Under laparoscopic visualization, two 8 mm robotic ports are placed superior and inferior to the camera port. Additional 12 and 5 mm port(s) can be placed below the two 8 mm robotic ports in the midline, superior and inferior to the umbilicus. The optional third 8 mm robotic port for the third robotic arm can be placed about three fingers above the ipsilateral anterior superior iliac spine 5 cm away from the inferior 8 mm robotic port. The robot cart is aligned and docked at an angle from behind the patient. The typical port site placement for left robot-assisted partial nephrectomy is illustrated in a diagrammatical sketch [Figure 1]. *(ii) Operative Dissection*, *Hilar Control and Renorraphy*: With a 30° down lens the colon is mobilised and reflected medially. Liver retraction can be provided by an assistant for right-sided renal tumours. The gonadal vein is identified along with the ureter and traced superiorly up to the renal area. The peri-renal fat is incised in the non-involved part of the kidney. Renal vein/artery is dissected by a combination of blunt dissection and electrocautery; vascular slings are placed around them as proximally as possible, and anchored with a Hem-o-lok™ (Teleflex, Research Triangle Park, NC) clip. A Surgicel™ (Ethicon Inc., Somerville, NJ) bolster and two 20 cm long Monocryl™ sutures [with a lapraTy™ (Ethicon, Cincinnati, OH,USA)/Hem-o-lok™ at its end] are placed inside the abdomen. Intraoperative laparoscopic ultrasound is performed via the 12 mm assistant port, to conform the extent and depth of the renal tumour. The tumour is then circumferentially scored with the right robotic hot monopolar forceps. Laparoscopic sponges soaked in distilled water may be placed alongside the renal tumour in order to minimise the risk of tumour implantation. An intravenous injection of 12.5 gm of Mannitol is administered prior to clamping of the renal vessels, in order to facilitate brisk intraoperative diuresis. Laparoscopic bulldog clips (Microfrance/Medtronic, Minneapolis, Minn) clamp(s) [LBD] are inserted to clamp the renal artery and the vein. The renal tumour is excised en-bloc with the overlying perinephric fat. In case of any doubt of whether the renal pelvicalyceal system has been entered, one can administer intravenous indigo carmine to visualise any gross leaks, in case these are apparent they can be repaired with a 7-8 inches long running suture of 3-0 vicryl^(™)^ on an RB-1 needle placed with the aid of the robotic needle driver and held in place with lapra-Ty^(™)^ clips. The renal defect can then be buttressed with a Surgicel™ bolster (only for wide defects exceeding 3-4 cm) and oversewn with 1-2 previously prepared 1-0 Monocryl™ sutures with a knot, and a preplaced LapraTy™ and a Weck Hem-o-Lock™ clip, proximal to its knotted end. Additional Hem-o-Lock™ clips can be placed by an assistant after centering the suture on the jaws of the clip, after each completed throw of the suture(s) which can be clipped and tightened (held with a ProGrasp™ forceps and slid down the renal capsule with a robotic needle driver). In case of persistent bleeding, an additional layer of Surgicel™ with 5 cc of FloSeal™ (Baxter Healthcare Corp, Fremont, CA, USA) can be applied to the renal defect. The laparoscopic bulldog clip remover is introduced via the 12 mm assistant port and the renal vessels are unclamped and the end of the warm ischemia time (WIT) is noted. The vascular slings are cut and removed along with the clip. After assuring haemostasis another injection of IV 12.5 gm Mannitol is administered. The perirenal fat is loosely re-approximated with a 3-0 Monocryl™ suture and the excised specimen is retrieved with an endoscopic bag. The robotic instruments are removed and the robot is undocked. *(iii) Closure*: A flank drain is generally placed in the renal bed and all the ports are removed under vision. The camera port is extended by about 1 cm to facilitate retrieval of the endoscopic bag. All port sites are closed at the fascia level with a Vicryl™ suture. The skin can be re-approximated with a sub-cuticular 4-0 Monocryl™ suture.

### Review of literature

Open partial nephrectomy has been the gold standard for <4 cm renal neoplasm(s), with the reported cure rates approaching close to those achieved with open radical nephrectomy.[2,3] The goal of RPN is to duplicate the oncological and functional outcome of open radical nephrectomy. The first laparoscopic partial nephrectomy (LPN) was performed through transperitoneal access and retroperitoneal access, by Winfield[4] and Gill *et al*.,[5] respectively. Robotic technique of partial nephrectomy was apparently initially performed and subsequently published by Gettman and colleagues in 2004.[6] With the emergence of the surgical daVinci^(™)^ robotic system, increasingly more workers[7–20] have also reported and described the technique of transperitoneal RPN [Table 1]. To date, the two largest series of RPN reported are by Rogers[13] and Wang *et al*.,[7] [Table 1].

**Table 1:** Salient features of the global robotic partial nephrectomy series^@^

Author/YR	No	BMIkg/m^2^	Tumour size (cm)	HC	ORT (mins)	WIT (mins)	EBL (ml)	HS (days)	FU (mths)	RCC	COMPLIC/CN (no)
Michli, 2009	20	28.5	2.7 (0.5-2.7)	-	142 (65-315)	28.1 (19-40)	263 (20-1600)	2.8	-	14/20	(3)-1CN,1RA,1 PE
Kaouk, 2009*	2	-	2.0	-	170 (113 -227)	-	100	3.5 (2.8-4.2)	-	2/2	-
Lee, 2009**	9	-	-	-	275 (170-417)	-	49 (5-150)	2.9 (1.9-4.8)	6 (0.2-25)	0	(2)-1U, 1PSI
Wang, 2009	40	29.7	2.4	LBD	141 (87-219)	20 (13-40)	137 (25-500)	2.5 (1-4)	NA	25/40	(1)-PM-1
Deane, 2008	10	-	3.1	LBD	228.7 (98-375)	32 (30-45)	115 (25-300)	2.0	16	7/10	(1)-BL
Aron, 2008	12	-	2.4	SC	242	23	329	4.7	7.4	9/12	-
Rogers, 2008a	08 (14 tumors)	-	2.4 (0.8-6.4)	LBD	192 (165-214)	31 (24-45)	230 (100-450)	2.6(2-3)	3	3/8	Nil
Rogers, 2008b˜	11+	-	3.8 (2.3-6.4)	-	202 (152-253)	28.9 (20-39)	220 (50-750)	2.6 (1-4)	-	11/11	(2)- 2UL
Rogers, 2008c^	148	-	2.8	-	197	27.8	183	1.9	-		(9)-3IL,2PE, 2UL,1BL,1RM
Ho, 2008a	25	-	3.1 (1.5-4.5)	-	82.6 (75-90)	22 (17-27)	122.6 (60-230)		-	16/25	-
Ho, 2008b	20	-	3.5 (2-5.5)	-	82.8 (75-95)	21.7 (15-27)	189 (50-260)	4.8 (4-7)	-	13/20	-
Kaul, 2007	10	-	2.3 (1.0-3.5)	LBD	155 (120-185)	21 (18-27)	92 (50-150)	1-2	15 (6-28)	8/10	(2)-1BL,1UL
Caruso, 2006	10	-	1.95	LBD	279	26.4	240	2.6	-	5/10	(4)-1UR, 2CN, 1PM
Philisp, 2005	12	-	1.8	LBD	265	26	240	2.7	-		(2)-1PM + UL1 REN
Gettman, 2004	13	-	2.5 (2.0-6.0)	LBD	215 (130-262)	22 (15-29)	170 (50-300)	4.3(2-7)	2-11	10/13	(2)-1PM,1IL
Total [Mean]@	350	-	2.4	-	191.27	25.37	162.64	2.95	-	67.95%	7.4%

˜ Used the four arm Robot (Tile Pro™)^+^,

+ Hilar tumours,

^ Denotes the largest Robotic PN series published to date.

* Single Port Robotic Cases,

** Pediatric series. TM - Tumour morphology, Ex - Exophytic, Ms - Mesophytic, En - Endophytic; RK - Right kidney, LK - Left kidney; HC - Hilar control, ORT - Operating room time; NT - Number of trocars; LBD - Laparoscopic bulldog clamps; SC - Satinsky clamps; HS - Hospital stay in days; FU - Follow up in months; Rcc - Renal cell carcinoma, PM - Positive margins; RA - Renal abscess; BL - Bleeding; U - Urinoma; PSI - Port site infection; UR - Urinary retention; IL - Ileus; UL - Urinary leak; PE - Pulmonary embolism; RM - Rhabdomyolysis; REN - Re-exploration nephrectomy; CN - Conversions to open/Hand assisted

Table 1 Shows a comparative review of the salient features of select series on robotic partial nephrectomy, as reported and published in the indexed English literature.

*(a) Tumour Characteristics*: The mean reported renal tumour size treated by RPN has been (2-3.1) cm[7–20] with the largest being 6.4 cm in size.[13] The use of intraoperative laparoscopic ultrasound and colour Doppler is useful for precise location, delineation of an endophytic tumour and for identifying the adjacent vessels.[10] Recently some have also used the TilePro^(™)^ (projection of preoperative CT images and live intraoperative ultrasound imaging directly on to the surgical console) feature of the daVinci-S^(™)^ robotic system to facilitate renal tumour localisation and resection.[21] In our opinion, for select endophytic or larger renal tumours in an otherwise inaccessible location, the use of an integrated, intraoperative, laparoscopic, ultrasound probe device like the TilePro^(™)^ can facilitate RPN. With the addition of the fourth arm for the robot it is apparent that this will provide the console surgeon a greater degree of precision in facilitating renal hilar dissection and vascular control. *(b) Hilar Control and Bleeding*: The challenging steps in RPN that require significant expertise and learning are hilar dissection/control and renorraphy. Hilar dissection/control is more daunting, as improper clamping due to overlooked accessory renal vessels can cause significant bleeding. This can be prevented by looking for immediate blanching of the kidney after clamping the renal hilar vessels, failure of the kidney to blanch should prompt the surgeon to release the clamps and dissect more proximally to reapply the laparoscopic bulldog clips. The presence of multiple renal vessels in about 20% of the cases is an important consideration (preoperative CT/MR angiography is an essential tool to evaluate them). Options include (i) enbloc clamping of all the renal vessels with a single Satinsky clamp or (ii) individual accessory vessel skeletonization and secure clamping with laparoscopic bulldog clips. Other workers have also demonstrated the feasibility of RPN in complex renal hilar tumors.[12] *(c) Haemostatic Agents*: Floseal™ is a synthetic thrombin-based gelatine matrix tissue hemosealant activated upon contact with blood, deliverable laparoscopically.[22] Risks of using Floseal™ include transmission of bovine spongiform encephalopathy/hepatitis (bovine source of collagen), but contact with urine hinders its action.[23] To alleviate the high cost of Floseal™ one may use the more economical purified potato starch powder (Arista™), which may turn out to be a promising substitute for haemostasis. Purified potato starch powder (PPSP) is composed of bio-polymeric, micro-porous polysaccharide hemospheres (MPH^®^) that acts as sieves, dehydrating the blood and accelerating the process of clotting. PPSP can be easily delivered through a 5 mm assistant laparoscopic port, using a distally fenestrated flexi tip applicator attached to a plastic billows container, it is also hypoallergenic and carries no risk of disease transmission.[24] 4 F.J. Murat, C.Q. Le and M.H. Ereth *et al*., Evaluation of microporous polysaccharide hemospheres for parenchymal hemostasis during laparoscopic partial nephrectomy in the porcine model, *JSLS* 10 (3) (2006), pp. 302-306. View Record in Scopus | Cited By in Scopus (2) Renorraphy with a Surgicel™ (oxidised wood pulp cellulose, Ethicon Inc., Somerville, NJ) bolster has been generally used to buttress renal defects following RPN, which acts by virtue of its cellulosic acid, producing an artificial clot due to its affinity for haemoglobin. It is a non-toxic, biocompatible, bio-soluble, bio-degradable anti-oxidant, with bactericidal and wound healing properties.[23] In our opinion and also of the others,[25] the use of Surgicel™ should be restricted to select small renal neoplasm(s), not easily amenable to a direct closure. Recently Weight and co-workers[26] described an alternative technique of no-bolstered renorraphy in LPN for centrally located tumours, to reduce their WIT. Experimental data has shown that the use of cold ischemia may be more advantageous for prolonged renal ischemia, and renal hypothermia may be associated with better haemostasis and renal function.[27] *(d) Outcome Parameters*: In the present series of published cases reviewed in Table 1 the mean ORT (CT) and hospital stay was 191.27 minutes and 2.95 days, respectively, in the global select RPN series [Table 1]. The computed average of WIT and estimated blood loss (EBL) has been 25.37 and 170 ml, respectively, in the global select RPN series [Table 1]. *(e) ORT and BMI*: Morbid obesity (body mass index greater than 30 kg/m^2^) may impact the results of RPN, especially in terms of the operating/console time and blood loss. Published data proves that morbid obesity may be related to a higher risk of renal cell carcinoma (RCC) and their surgical management may be associated with higher or similar complication rates.[28,29] In our opinion obesity may no longer be viewed as a contraindication to RPN. *(f) WIT and Renal function*: In our opinion certain measures can be adopted in select cases to further reduce the WIT, and/or maximise the renal function including: (i) no bolster renorraphy for select centrally located renal tumours, (ii) bolstered renorraphy for polar tumours with suturing of the collecting system, (iii) routine use of the sliding clip renorraphy technique, (iv) maximising the use of TilePro™ and the fourth arm of the robot during an RPN and (v) considering the use of cold ischemia in place of warm ischemia. Mean WIT has been 25.37 minutes in the global RPN series [Table 1]. Others have reported a ‘U’ looped vascular sling passed around the renal vessels, threaded over a tube, at its ends (that functions as a tourniquet), to enable a transient vascular occlusion in the RPN, as an alternative to the LBD/Satinsky™ clamp.[30] Some workers have used the fourth arm of the robot with its long tip forceps to occlude the renal artery during resection of the renal mass in an RPN.[31] However it remains to be proven whether this actually has the potential to lower WIT and improve renal function. *(g) Complications*: Review of a select series reveals an overall 7.4% complication rate associated with RPN, most of which appears to be minor in nature. *Minor*: Lee *et al*.,[17] reported port site infection in 1/9 patients. Michli *et al*.,[20] reported the detection of renal abscess in 1/20 of their patients. Getman *et al*.,[6] reported prolonged ileus in 1/13 of their patients following RPN. Urinary leak following RPN was reported by Roger *et al*.[12,13] and Kaul *et al*.,[11] in 2/11-2/148, and 1/10 of their patients respectively. Lee *et al*.,[17] also reported the occurrence of a urinoma in 1/9 of their patients. *Major*: Michli[20] and Caruso *et al*.,[14] reported a conversion to open/hand-assisted laparoscopic partial nephrectomy in 3/20 and 2/10 patients undergoing a planned RPN procedure. Excessive troublesome bleeding associated with RPN was reported by Dean *et al*.,[8] Rogers *et al*.,[13] and Kaul *et al*.,[11] in 1/10, 1/148, and 1/9 of their patients, respectively. Wang *et al*.,[7] Philisp *et al*.[15] and Getman *et al*.,[6] also reported a positive tumour margin (PSM) in 1/40, 2/12 and 2/13 patients undergoing RPN, respectively. Michli *et al*.[20] and Gettman *et al*.,[6] also reported the occurrence of pulmonary embolism in 1/20 and 2/144 of their patients undergoing RPN.

According to a certain comparative series[32,33] the mean ORT, EBL and WIT may be significantly lower with RPN versus LPN, however, individual results tend to vary. Larger prospective randomised controlled trials between the two are the need of the day in order to properly address this issue. Cost factor may serve as a drawback to the universal acceptance of RPN for T1 renal tumours; while no cost comparisons have been reported in the literature when comparing the cost of RPN versus LPN, it likely that due to the high recurring cost of the da-Vinci^(™)^ surgical robotic system, the former would be more expensive.[34] RPN is overall a complex surgical procedure that should be undertaken by urologists familiar with the technique of open and/or laparoscopic partial nephrectomy and who are well-conversant with the technicalities of robotic console surgery. Excellent coordination between the robotic console surgeon, assistant surgeon and other team members is of paramount importance in order to achieve a consistently favourable outcome.

## CONCLUSIONS

In our opinion robot-assisted laparoscopic partial nephrectomy is a therapeutically adequate, minimally invasive, surgical modality of choice for most cases of incidental and early renal neoplasm(s) of T_1_ stage. More than 350 cases of RPN have been currently performed worldwide; it is currently the most minimally invasive alternative surgical modality of choice to open/laparoscopic partial nephrectomy that best mimics the oncological principles of surgery. With adequate experience, select multiple, complex, hilar and peri-hilar renal lesions too can be treated successfully with RPN. Morbid obesity may not be associated with a longer operating time. The drawbacks of some of the currently published studies reviewed in this manuscript include lack of renal scans, analgesic requirements and pain scores; these were not considered, as also long-term data are still awaited. Few studies have evaluated a comparative assessment of RPN with LPN.

## References

[1] Luciani LG, Cestari R, Tallarigo C (2000). Incidental renal cell carcinoma-age and stage characterization and clinical implications: Study of 1092 patients (1982-1997). Urology.

[2] Lee CT, Katz J, Shi W (2000). Surgical management of renal tumours 4 cm or less in a contemporary cohort. J Urol.

[3] Novic C, Derweesh I (2005). Open partial nephrectomy for renal tumors: Current status. BJU Int.

[4] Winfield HN, Donovan JF, Godet AS, Clayman RV (1993). Laparoscopic partial nephrectomy: Initial case report for benign disease. J Endourol.

[5] Gill IS, Delworth MG, Munch LC (1994). Laparoscopic retroperitoneal partial nephrectomy. J Urol.

[6] Gettman MT, Blute ML, Chow GK, Neururer R, Bartsch G, Peschel R (2004). Robotic-assisted laparoscopic partial nephrectomy: Technique and initial clinical experience with da Vinci robotic system. Urology.

[7] Wang AJ, Bhayani SB (2009). Robotic partial nephrectomy versus laparoscopic partial nephrectomy for renal cell carcinoma: Single-surgeon analysis of >100 consecutive procedures. Urology.

[8] Deane LA, Lee HJ, Box GN, Melamud O, Yee DS, Abraham JB (2008). Robotic versus standard laparoscopic partial/wedge nephrectomy: A comparison of intraoperative and peri-operative results from a single institution. J Endourol.

[9] Aron M, Koenig P, Kaouk JH (2008). Robotic and laparoscopic partial nephrectomy: A matched-pair comparison from a high volume centre. BJU Int.

[10] Rogers CG, Singh A, Blatt AM (2008a). Robotic partial nephrectomy for complex renal tumors: Surgical technique. Eur Urol.

[11] Kaul S, Laungani R, Sarle R (2007). da Vinci-assisted robotic partial nephrectomy: Technique and results at a mean of 15 months of follow-up. Eur Urol.

[12] Rogers CR, Metwalli A, Blatt AM, Bratslavsky G, Menon M, Linehan WM (2008b). Robotic partial nephrectomy for renal hilar tumors: A multi-institutional analysis. J Urol.

[13] Rogers CG, Menon M, Weise ES (2008c). Robotic partial nephrectomy: A multi-institutional analysis. J Robot Surg.

[14] Caruso RP, Phillips CK, Kau E (2006). Robot-assisted laparoscopic partial nephrectomy: Initial experience. J Urol.

[15] Phillips CK, Taneja SS, Stifelman MD (2005). Robot-assisted laparoscopic partial nephrectomy: The NYU technique. J Endourol.

[16] Kaouk JH, Goel RK (2009). Single-port laparoscopic and robotic partial nephrectomy. Eur Urol.

[17] Lee RS, Sethi AS, Passerotti CC, Retik AB, Borer JG, Nguyen HT (2009). Robot-assisted laparoscopic partial nephrectomy: A viable and safe option in children. J Urol.

[18] Ho HS, Peschel R, Neururer R, Steiner H, Schwentner C, Bartsch G (2008a). Another novel application of hem-o-lok clips for transient vascular occlusion in robot-assisted laparoscopic partial nephrectomy: An alternative to laparoscopic bulldog and satinsky clamps. J Endourol.

[19] Ho H, Schwenter C, Neururer R, Steiner H, Bartsch G, Peschel R (2008b). Robotic assisted laparoscopic partial nephrectomy: Surgical technique and clinical outcomes at one year. BJU Int.

[20] Michli EE, Parra RO (2009). Robotic-assisted laparoscopic partial nephrectomy: Initial clinical experience. Urology.

[21] Rogers CR, Laungani R, Bhandari A, Krane LS, Eun D, Patel MN (2009). Maximizing console surgeon independence during robot-assisted renal surgery by using the fourth arm and tilepro™. J Endourol.

[22] Walters RC, Collins MM, L'Esperance JO (2006). Hemostatic techniques during laparoscopic partial nephrectomy. Curr Opin Urol.

[23] Singh Iqbal, Saran RN, Jain M (2009). Does sealing of the tract with absorbable gelatin (Spongostan) facilitate tubeless PCNL?. A prospective study. J Endourol.

[24] Murat FJ, Le CQ, Ereth MH (2006). Evaluation of microporous polysaccharide hemospheres for parenchymal haemostasis during laparoscopic partial nephrectomy in the porcine model. Journal of the Society of Laparoendoscopic Surgeons.

[25] Agarwal D, O'Malley Clarke D, Rao R (2007). Modified technique of renal defect closure following laparoscopic partial nephrectomy. BJU Int.

[26] Weight CJ, Lane BR, Gill IS (2007). Laparoscopic partial nephrectomy for selected central tumors: Omitting the bolster. BJU Int.

[27] Constantinides CA, Tyritzis SI, Evangelou C, Kyroudi A, Liatsikos E, Karamessinis P (2008). Vascular endothelial growth factor protein expression in a renal ablation rabbit model under prolonged warm and cold ischemia. Am J Nephrol.

[28] Anast W, Stoller ML, Meng MV (2004). Differences in complications and outcomes for obese patients undergoing laparoscopic radical, partial or simple nephrectomy. J Urol.

[29] Colombo JR, Haber GP, Aron M, Xu M, Gill IS (2007). Laparoscopic partial nephrectomy in obese patients. Urology.

[30] Ho SH, Peschel R, Neurer R, Steiner H, Schwenter C, Bartsch G (2008c). Another novel application of Hem-o-Lok clips for transient vascular occlusion in robot-assisted laparoscopic bulldog and satinsky clamps. J Endourol.

[31] Figenshau R, Bhayani S, Venkatesh R, Wang A (2008). Robotic hilar control and robotic clip placement for partial nephrectomy. J Endourol.

[32] Kural AR, Atug F, Tufek I, Akpinar H (2009). Robot-assisted partial nephrectomy versus laparoscopic partial nephrectomy: Comparison of outcomes. J Endourol.

[33] Benway BM, Bhayani SB, Rogers CG, Dulabon LM, Patel MN, Lipkin M (2009). Robot-assisted partial nephrectomy versus laparoscopic partial nephrectomy for renal tumors: A multi-institutional analysis of perioperative outcomes. J Urol.

[34] Gautam G, Benway BM, Bhayani SB, Zorn KC (2009). Robot-assisted partial nephrectomy: Current perspectives and future prospects. Urology.

